# Epidemiology and clinical features of respiratory syncytial virus (RSV) infection in hospitalized children during the COVID‐19 pandemic in Gorgan, Iran

**DOI:** 10.1002/hsr2.1787

**Published:** 2024-01-03

**Authors:** Mahnaz Ramzali, Vahid Salimi, Fatemeh Cheraghali, Seyedeh Delafruz Hosseini, Mohammad Yasaghi, Saeed Samadizadeh, Mostafa Rastegar, Britt Nakstad, Alireza Tahamtan

**Affiliations:** ^1^ Infectious Diseases Research Center Golestan University of Medical Sciences Gorgan Iran; ^2^ Department of Microbiology, School of Medicine Golestan University of Medical Sciences Gorgan Iran; ^3^ Department of Virology, School of Public Health Tehran University of Medical Sciences Tehran Iran; ^4^ Department of Pediatrics, School of Medicine, Taleghani Children's Hospital Golestan University of Medical Sciences Gorgan Iran; ^5^ Department of Pediatric and Adolescent Health University of Botswana Gaborone Botswana; ^6^ Division of Paediatric and Adolescent Medicine, Institute of Clinical Medicine University of Oslo Oslo Norway

**Keywords:** COVID‐19, influenza, polymerase chain reaction, respiratory syncytial virus

## Abstract

**Background and Aims:**

Respiratory syncytial virus (RSV) is a leading cause of acute respiratory infection in infants and young children. Given the altered circulation patterns of respiratory viruses during the coronavirus disease pandemic‐2019 (COVID‐19), the study aimed to evaluate epidemiology and clinical features of RSV infections in hospitalized children during the COVID‐19 pandemic in Gorgan, northeastern Iran. Molecular epidemiology studies on respiratory viral infections are necessary to monitor circulating viruses, disease severity, and clinical symptoms, in addition to early warning of new outbreaks.

**Methods:**

Overall, 411 respiratory swab samples from hospitalized children from October 2021 to March 2022 were collected at Taleghani Children's Hospital, Gorgan, Iran. The incidence of RSV, as well as the circulating subgroups and genotypes, were investigated and confirmed using PCR methods. Additionally, all samples tested for severe acute respiratory syndrome‐associated coronavirus 2 (SARS‐CoV‐2) and influenza, and demographic and clinical data were analyzed using SPSS software.

**Results:**

The share of RSV, SARS‐CoV‐2, and influenza among hospitalized children with acute lower respiratory infections (ALRI) were 27%, 16.5%, and 4.1%, respectively. The RSV subgroup A (genotype ON1) was dominant over subgroup B (genotype BA9), with more severe clinical symptoms. Compared with the prepandemic era there were high numbers of hospitalized SARS‐CoV‐2 positive children and low numbers of other respiratory viruses. Despite this, the prevalence of ALRI‐related RSV‐disease among hospitalized children in our specialized pediatric center was higher than COVID‐19 disease in the same cohort.

**Conclusions:**

Studying the epidemiology of respiratory viruses and determining the circulating strains can contribute to effective infection control and treatment strategies.

## 1 INTRODUCTION

Respiratory syncytial virus (RSV) causes infections of the lungs and respiratory tract. It is so common that most children have been infected with the virus by age 2. Symptoms of RSV infection vary from mild upper respiratory tract infections to acute lower respiratory tract infection (ALRI), such as bronchiolitis and pneumonia.^1^, ^2^ Globally RSV disease, among hospitalized children under 5, has been estimated to account for 28% of overall ALRI with high (13%–22%) mortality rates. More than 93% of episodes and 99% of the deaths occur in developing countries.^3^ The risk of severe RSV‐disease is highest in premature infants and those with chronic lung disease, congenital heart defects, and immunodeficiency disorders.^2^, ^4^ Severe early‐life RSV‐disease may even predispose some infants to develop childhood asthma.^5^

There are two main subtypes, RSV A and B, with different genotypes circulating throughout the year,^6^ with a seasonal pattern in temperate climates.^6^, ^7^ To develop and plan strategies for prevention or reducing RSV‐associated child morbidity and mortality it is important to understand the epidemiology and the burden of RSV infection.^8^ Understanding the risk factors for severe infection allows us to identify high‐risk groups and provide more efficient patient management.^4^, ^9^ In Iran, from 1996 to 2013, the prevalence of RSV‐disease was reported to be 18.7%, with the highest number of positive cases between November and March. More recently, from 2018 to 2019, two genotypes of RSV, ON1 (RSV‐A) and BA9 (RSV‐B), were most prevalent in viral isolates.^10^ During the coronavirus disease pandemic‐2019 (COVID‐19), the circulation of other respiratory viruses changed, probably due to health and care measures taken to prevent severe acute respiratory syndrome‐associated coronavirus 2 (SARS‐CoV‐2) disease. In many countries, RSV surprisingly disappeared between 2020 and 2021, possibly due to lockdown and precautions taken to fight the COVID pandemic.^11^, ^12^ Due to varying reports about RSV spread during the COVID‐19 pandemic, we aimed to investigate the prevalence and clinical features of RSV‐disease in infants and children in Golestan province, north of Iran, during the COVID pandemic.

## 2 MATERIALS AND METHODS

Between October 2021 and March 2022, we recruited 411 hospitalized Iranian children with viral induced ALRI including sneezing, cough, dyspnea, and fever at Taleghani Children's Hospital, Gorgan, Iran. The hospital is the only specialized pediatric center in Golestan province. The samples were collected during the COVID‐19 pandemic and examined for SARS‐CoV‐2 and influenza performed at the Golestan province coronavirus laboratory.^13^, ^14^ Demographic and clinical information of the patients were collected by the caregivers and relatives of the patients in a questionnaire. This study was approved by the science and bioethics committee of Golestan University of Medical Sciences (IR.GOUMS.REC.1401.023).

**Figure 1:**
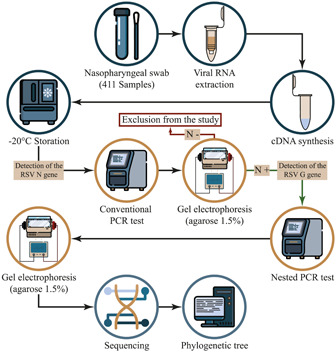
Flowchart showing the procedure of study.

Flowchart of the study is shown in Figure 1. For the RSV detection test, viral RNA was extracted from nasopharyngeal swabs using a viral high pure nucleic acid extraction kit following the manufacturer's instructions (BehPrep Viral RNA Extraction kit). The extracted RNA was converted into cDNA using the cDNA reverse transcription kit (Yekta Tajhiz Azma) according to the manufacturer's instructions.

The presence of the RSV genome (N gene) in collected samples was analyzed through the conventional PCR method using specifically designed primers (Table 1). The cDNA (100 ng) was added to 20 μL of reaction mixtures containing optimal buffers, each deoxynucleoside triphosphate at a final concentration of 200 μM, 3.0 mM MgCl2, 0.5 μM forward and reverse primers, and 0.5 units of Taq DNA Polymerase. The reaction conditions were: 95°C for 10 min followed by 35 cycles of 95°C for 30 s, 56°C for 30 s, 72°C for 1 min, and then 72°C for 5 min. Finally, the amplified product was analyzed by electrophoresis on 1.5% agarose.

**Table 1:** Selected primers for amplification of viral RNA from RSV A and B.

Primers	Sequence	Location	Tm	Product size
RSV‐N	F:5′‐ATGGCTCTTAGCAAAGTCAAGT‐3'	1085‐1106	57.97	122 bp
	R: 5′‐TGCACATCATAATTRGGAGTRTCA‐3'	1206‐1183	55.49	
RSV‐A: Outer	F: 5′‐ AGTGTTCAACTTTGTACCCTGC‐3′	5120‐5141	59.7	594 bp
	R: 5′‐ CTGCACTGCATGTTGATTGAT‐3′	5713‐5693	58	
RSV‐A: Inner	F: 5′‐ CACCACCAAGCCCACGAAA ‐3′	5171‐5189	60.83	454 bp
	R: 5′‐ ATTGTTATTTGCCCCATAGTTT‐3′	5624‐5603	56.34	
RSV‐B: Outer	F: 5′‐ GCAGCCATAATATTCATCATCTCT‐3′	4801‐4824	56.75	855 bp
	R: 5′‐ TGCCCCAGRTTTAATTTCGTTC‐3′	5655‐5634	57.29	
RSV‐B: Inner	F: 5′‐ ACAAACAAACYACCCACCAAA ‐3′	5246‐5266	59.54	400 bp
	R:5′‐ TGCTTGAGGGATCAYRGTTC ‐3′	5645‐5626	60.55	

The positive samples were tested for the RSV‐G gene using nested PCR. Primers were designed to detect the RSV‐A and B strains (Table 1). In both steps of the nested PCR for RSV‐A and RSV‐B, 100 ng of the sample was added to 20 μL of reaction mixtures containing optimal buffers. The reaction conditions for RSV‐A were as follows: for the outer stage, 95°C for 5 min followed by 35 cycles of 95°C for 1 min, 64.7°C for 1 min, 72°C for 1 min, and then 72°C for 5 min; for the inner stage, 95°C for 5 min followed by 35 cycles of 95°C for 1 min, 62°C for 1 min, 72°C for 1 min, and then 72°C for 5 min. The reaction conditions for the outer stage of RSV‐B were as follows: 95°C for 10 min followed by 40 cycles of 95°C for 30 s, 58°C for 30 s, 72°C for 1 min, and then 72°C for 5 min; for inner stage, 95°C for 10 min followed by 40 cycles of 95°C for 30 s, 56°C for 30 s, 72°C for 1 min, and then 72°C for 5 min. Finally, the amplified product was analyzed by electrophoresis on 1.5% agarose. RSV‐A and B positive samples were sequenced and results were analyzed via the Sanger method and Chromass 2.6.6. software. The phylogenetic tree was drawn by Mega7 software using the Maximum likelihood method with 1000 bootstrap replicates.

The collected data were analyzed using SPSS software. Results were presented as mean and standard deviation and qualitative data as frequency (percentage). *χ* ^2^ and Fisher's tests were used to perform statistical analysis. The *χ* ^2^ test was used with a large sample size and expected frequency counts greater than 5, while Fisher's exact test was used when the sample size was small, and the expected frequency counts were less than 5. Significant correlations were considered if *p* values less than 0.05. The STROBE checklist was followed in the manuscript (https://www.equator-network.org/reporting-guidelines/strobe/).

## 3 RESULTS

Among the 411 samples, 252 (61.3%) and 159 cases (38.7%) were male and female, respectively. The average age of the patients was 20.7 ± 19.3 months, and the highest number of patients was between 6 and 24 months.

**Figure 2:**
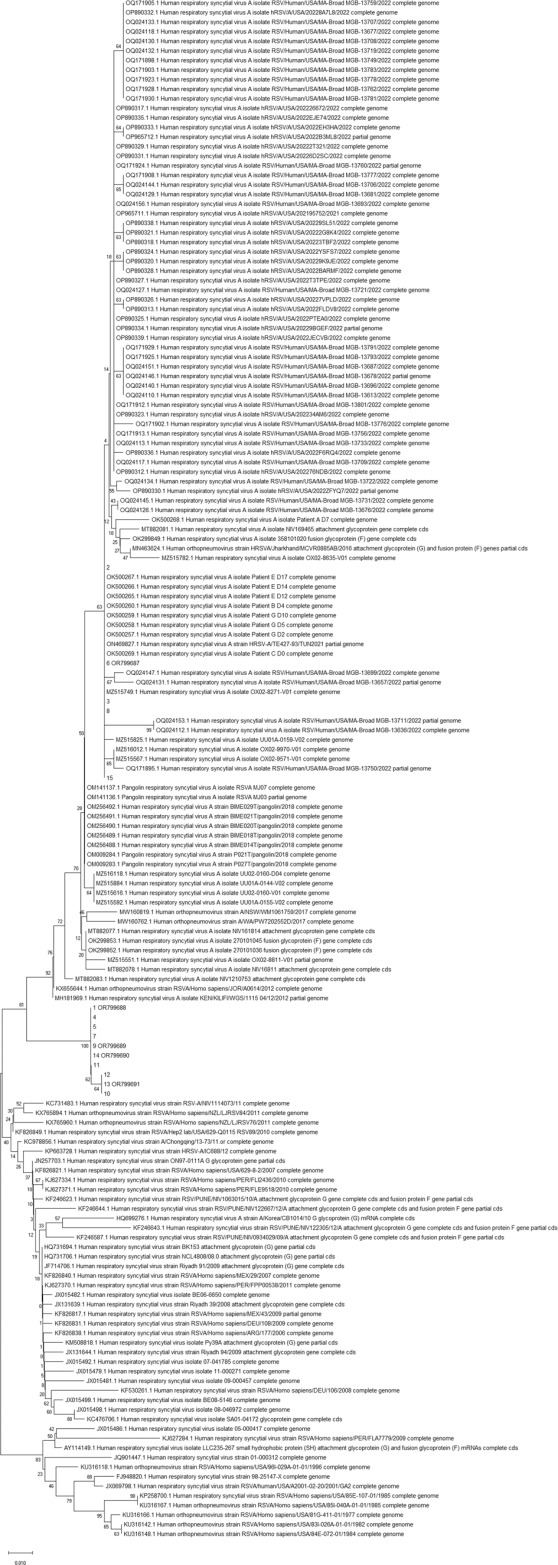
The phylogenetic tree drawn by Mega7 software using the Maximum likelihood method with 1000 bootstrap replicates.

Out of 411 samples, 111 were identified with RSV‐N gene primers and considered as positive RSV patients (Supporting Information: Figure 1). Upon analysis of the RSV‐G gene, the frequency of the RSV was 27%, of which 60.4% (67 cases) were subtype A (Supporting Information: Figure 2) and 14.3% (16 cases) were subtype B (Supporting Information: Figure 3). Moreover, 25.3% (*n* = 28) of positive cases were not identified, possibly due to the low titer of the virus in these samples. The positive cases of both subgroups A and B were explored with sequencing. All RSV‐A cases belonged to the ON1 genotype (GenBank accession numbers: OR799687, OR799688, OR799689, OR799690, OR799691), and all RSV‐B cases belonged to the BA9 genotype (GenBank accession number: OR799692). The phylogenetic tree is shown in Figure 2. The frequency of SARS‐CoV‐2 and influenza was 68 (16.5%) and 17 (4.1%), respectively. Eight (7.2%) cases were positive for both RSV and SARS‐CoV‐2 and one (0.9%) were positive for both RSV and influenza.

There was a nonsignificant gender inequality in the 111 positive samples, 66 (59.5%) were boys, and 45 (40.5%) were girls (Table 2). A significant difference was observed between age‐groups and RSV‐positivity (*p* < 0.05). RSV‐positive cases were predominantly children aged 6–24 months.

**Table 2:** Distribution of RSV subgroups and genotypes and demographic details for patients infected with RSV.

Variable	RSV	*p* Value	RSV‐A	*p* Value	RSV‐B	*p* Value
*Gender*						
Male	66 (59.5%)	0.63	41 (61.2%)	0.98	8 (50%)	0.34
Female	45 (40.5%)		26 (38.8%)		8 (50%)	
*Age (month)*						
0–2	7 (6.3%)	**0.003**	4 (6%)	**0.01**	2 (12.5%)	0.45
2–6	36 (32.4%)		24 (35.8%)		5 (31.3%)	
6–24	40 (36%)		23 (34.3%)		6 (37.5%)	
24–60	21 (18.9%)		13 (19.4%)		3 (18.8%)	
60–86	7 (6.3%)		3 (4.5%)		0	

Symptoms in positive cases were primarily cough and fever, with more difficulty of breathing, wheezing, as well as lethargy (*p* < 0.05). There was a significant correlation between subgroup A and cough, wheezing, and shortness of breath, and in subgroup B with cyanosis (both *p* < 0.05). Out of 111 RSV‐positive cases, 28 cases (25.2%) had an underlying disease (Table 3), but no significant correlation was found between RSV‐positivity and their underlying disease. Ten positive cases (9%) were born preterm, but without a significant correlation to severity of symptoms.

**Table 3:** Distribution of RSV subgroups and multivariable details for patients infected with RSV.

Characteristics	Total (%)	RSV (%)	*p* Value	RSV‐A (%)	*p* Value	RSV‐B (%)	*p* Value
*Clinical symptoms*							
Cough	312 (75.9)	90 (81.1)	0.13	60 (89.6)	**0.00**	15 (93.8)	0.13
Fever	305 (74.2)	79 (71.2)	0.39	46 (68.7)	0.25	10 (62.5)	0.25
Difficult breath	115 (28)	40 (36)	**0.02**	24 (35.8)	0.11	5 (31.3)	0.77
Wheezing	112 (27.3)	40 (36)	**0.01**	26 (38.8)	**0.02**	7 (43.8)	0.15
Lethargy	129 (31.4)	26 (23.4)	**0.03**	15 (22.4)	0.08	2 (12.5)	0.09
Vomit	109 (26.5)	24 (21.6)	0.17	13 (19.4)	0.14	3 (18.8)	0.57
Hysteria	59 (14.4)	16 (4.4)	0.98	5 (7.5)	0.07	2 (12.5)	1
Fast breath	31 (7.5)	13 (11.7)	**0.05**	10 (14.9)	**0.01**	2 (12.5)	0.34
Cyanosis	26 (6.3)	10 (9)	0.32	2 (35)	0.42	5 (31.3)	**0.00**
Chest pain	4 (1)	1 (0.9)	0.38	1 (1.5)	0.81	0	–
*Underlying diseases*							
Neuromuscular disease	36 (8.8)	9 (1.8)	0.77	3 (4.5)	0.17	0	–
Icterus	25 (6.1)	6 (5.4)	0.72	3 (4.5)	0.78	2 (12.5)	0.25
Chronic heart disease	11 (2.7)	2 (1.8)	0.22	7 (10.4)	1.77	0	–
Congenital heart disease	11 (2.7)	2 (1.8)	0.73	0	–	1 (6.3)	0.35
Blood disease	6 (1.5)	2 (1.8)	0.66	2 (3)	0.25	0	–
Kidney disease	5 (1.2)	3 (2.7)	0.12	1 (1.5)	0.59	0	–
Genetic disease	3 (0.7)	1 (0.9)	1	0	–	0	–
Respiratory failure	3 (0.7)	1 (0.9)	1	0	–	0	–
UTI	5 (1.2)	1 (0.9)	1	0	–	0	–
Down syndrome	2 (0.5)	1 (0.9)	0.46	0	–	0	–
*Premature*	40 (9.7)	10 (9)	0.65	5 (7.5)	0.64	1 (6.3)	0.85
*Respiratory involvement*							
Pneumonia	231 (56.2)	73 (65.8)	**0.01**	46 (68.7)	**0.02**	11 (68.8)	0.3
Bronchiolitis	37 (9)	12 (10.8)	0.43	9 (13.4)	0.16	1 (6.3)	1
Asthma	11 (2.7)	3 (2.7)	1	1 (1.5)	1	1 (6.3)	0.35
*Vaccination*							
Complete vaccination	357 (86.9)	91 (82)	**0.02**	57 (85.1)	0.3	12 (75)	0.68
Incomplete vaccination	52 (12.7)	18 (16.2)		10 (14.9)		4 (25)	
Unknown	2 (0.5)	2 (1.8)		0		0	
*SPO2*							
>93%	347 (84.4)	85 (76.6)	**0.02**	52 (77.6)	0.23	11 (68.8)	0.19
<93%	54 (13.1)	21 (18.9)		13 (19.4)		4 (25)	
Unknown	10 (2.4)	5 (4.5)		2 (3)		1 (6.3)	
*Medical support*							
Ventilation	136 (33.6)	47 (42.3)	**0.02**	30 (44.8)	**0.03**	6 (37.5)	0.73
*Disease severity*							
ICU	34 (8.3)	10 (9)	0.74	4 (6)	0.45	1 (6.3)	1
*The outcome of the disease*							
Death	8 (1.9)	1 (0.9)	0.68	1 (1.5)	1	0	**–**

*Note*: The *χ* ^2^ test was used when the sample size was large, and the expected frequency counts were greater than 5, while Fisher's exact test was used when the sample size was small, and the expected frequency counts were less than 5.

Among the positive cases, 73 (65.8%) had pneumonia, related to RSV infection and subtype A. Among the hospitalized children there was a significant correlation between those with incomplete (vs. complete) routine vaccination and RSV disease. Eighty‐one percent (91/111) and 18/111 (16.2%) had full and incomplete vaccination, respectively. Oxygen saturations (SpO2) at admittance or during stay were below 93% in 25% of the positive RSV cases, indicating an important correlation of RSV disease with low SpO_2_. Forty‐seven (42.3%) of the RSV‐positive patients needed mechanically assisted ventilation. We found a significant correlation between RSV‐positivity and ventilator treatment, as well subgroup A and ventilator treatment. Ten (9%) were hospitalized in our intensive care unit (ICU) and one patient (0.9%) demised. He was a 7‐month‐old male with spinal muscular atrophy (SMA) and chronic heart disease. His initial respiratory symptoms included cough, fever, shortness of breath, wheezing. He was diagnosed with pneumonia needing ventilator treatment in the intensive care unit due low SpO2 and respiratory distress.

## 4 DISCUSSION

This study aimed to evaluate the epidemiology and clinical features of RSV infection in hospitalized children during the COVID‐19 pandemic in Gorgan, northeastern Iran. The prevalence of RSV‐disease, as well as the circulating subgroups and genotypes, were investigated and confirmed using PCR methods. During the pandemic, RSV‐disease among hospitalized children was more prevalent than that of SARS‐CoV‐2 and influenza disease, with predominant circulating genotype RSV‐A ON1 over RSV‐B genotype BA9. Epidemiological knowledge about respiratory viruses and how they circulate, when they cause disease, the severity and symptoms seem important for prevention and mitigation of new outbreaks following the COVID‐19 pandemic.

Respiratory infections can affect children at any time of the year, and is more common in temperate climates.^15^ In our study, the highest number of cases was observed in January and February 2022 (winter season), similar to Malekshahi et al.^16^ This was seen despite worldwide reports of delayed or altered seasonality of RSV infection in several studies during the pandemic.^12^, ^17^, ^18^ Seasonal variations in respiratory viral infections may be related to children staying indoors more often in cold seasons and being exposed to others who may be infected. Moreover, many viruses thrive in low humidity, which makes the nasal passages drier and more susceptible to infection.^19^

In this study, more RSV‐disease was seen in hospitalized children compared with SARS‐CoV‐2 and influenza. In contrast, several other studies reported that the prevalence of RSV disease decreased dramatically after the emergence of SARS‐CoV‐2.^20^, ^21^, ^22^ A study in the United States found that RSV circulation was historically low during 2020–2021, began earlier and continued longer during 2021–2022 compared with prepandemic years and seasons.^23^ In China RSV disease showed atypical seasonality during the pandemic, with a peak incidence in May 2020 and a second peak in November 2020, which was different from the typical winter peak observed in previous years.^24^ In Australia, RSV disease was significantly reduced compared with prepandemic years, with no detectable RSV activity from April to September 2020. The RSV peak appeared later than in prepandemic years, from October to December 2020.^25^

In Iran, Malekshahi et al. reported an overall 46% prevalence of RSV‐disease in December 2015 to April 2016, among all cases of ALRI less than 2 years,^16^ while in the winter season of 2021–2022 we found 27% with RSV‐disease among children hospitalized with ALRI. In Italy, during the COVID pandemic, 1.6% of 1213 samples was positive for RSV, with subgroups A (44.4%) and B (55.6%). The peak occurred in December 2021, when the RSV prevalence was 4.6%. All RSV A and B strains belonged to the ON1 and BA genotypes, respectively.^26^ In Japan, comparing RSV activity in 2021 with four previous seasons, they proved a prevalence of 56.8% RSV‐positive cases aged ≥2 years, higher than the 31.2% reported in the past 5 years. The study also suggested that infants aged <1 year, supposed to be especially susceptible to RSV infection, were less likely to be infected with RSV because of COVID‐19 control measures.^27^ This reduction may be due to public health and social measures such as facial coverings, mask‐wearing, hand hygiene, social distancing, and closure of kindergartens and schools, as well as improved surveillance and response measures. Another explanation could be competition and interference between viruses, although some studies reported high rates of co‐infections between RSV and SARS‐CoV‐2, which might challenge this hypothesis.^28^, ^29^, ^30^

The prevalence of coinfections varies from country to country. Among hospitalized children, we report 7% RSV‐positive cases with co‐infections of RSV and SARS‐CoV‐2, while only a single case (0.9%) was detected with both RSV and influenza. According to a retrospective review study in United States 2021, only 1.4% were co‐infected with RSV and SARS‐CoV‐2,^31^ while in UK, between February 2020 and December 2021, 3.2% were co‐infected with RSV and SARS‐CoV‐2.^32^ Studies from Italy, Poland, and South Africa did not report such co‐infections,^33^, ^34^, ^35^ however, in Iran and Brazil they found high rates of co‐infections with SARS‐CoV‐2 and influenza, ranging from 9.7% to 22.3%.^36^, ^37^ We speculate that co‐infections with RSV and other respiratory viruses may increase the risk of developing severe acute respiratory infections.^6^, ^38^ However, we proved no significant correlation between RSV‐related ALRI severity and other respiratory viruses such as Rhinovirus, Enterovirus, and Metapneumovirus, possibly due to the small sample size.

In our cohort RSV subtypes A and B were prevalent, with subtype A being predominant with or without co‐infection. These findings are consistent with reports from studies both during the COVID‐19 pandemic and in the prepandemic era, suggesting a novel strain‐related outbreak after lifting preventive measures and restrictions.^28^ In this study, all RSV‐A and B cases belonged to the ON1 and BA9 genotypes, respectively, consistent with Malekshahi et al. (2015–2016) and Tavakoli et al. (2018–2019) in Tehran, Iran.^10^, ^16^ Also in Saudi Arabia, all positive RSV‐A cases belonged to the ON1 genotype, while all positive RSV‐B cases belonged to the BA9 genotype.^39^ The ON1 and BA9 genotypes contain 72 and 60 bp in the second variable region of the G gene, respectively. This lengthening modification in glycoprotein G is likely an evolutionary advantage that could be the reason for the dominance of these genotypes. In addition, glycosylation of the C‐terminal end of the G protein can affect the expression of epitopes and hide them from detection by antibodies.^40^, ^41^

The RSV disease severity varies among individuals due to several virus‐ and host‐associated factors.^42^, ^43^ In this study the relationship between RSV disease severity and subgroups, indicate that severity was associated with subtype A. Significant correlations was seen between RSV‐A positivity and cough, wheezing, shortness of breath, and respiratory distress among the hospitalized children. Previous studies confirmed these severe symptoms in RSV disease, but without a significant correlation between RSV disease severity and subgroups.^41^, ^44^ Furthermore, there was a significant correlation between hospitalized RSV‐positive cases and pneumonia, whereas no correlation was seen with bronchiolitis, in contrast to Tabatabaei et al. and Tran et al.^41^, ^45^ Additionally, we proved a significant correlation between RSV subgroup A, and low SpO2 levels with a need for mechanical ventilation, in accordance with a study from Brazil by Vianna et al.^46^

The number of RSV‐infected individuals was higher than those infected with SARS‐CoV‐2 and influenza. In accordance with Tran et al.,^45^ the majority of RSV‐diseased children were between 6 and 24 months. This underscores the vulnerability and severity of RSV disease in the youngest children, in line with others^41^, ^47^, ^48^ and may explain why RSV infections, especially subgroup A, are more prominent in this cohort compared with SARS‐CoV‐2 and influenza. Several studies have indicated a higher prevalence of RSV disease in males.^6^, ^12^ In contrast, we found no gender differences, but RSV infection tended to be more prevalent in males than females, with predominance of subtype A, whereas RSV subtype B were equally distributed among male and females.

Children with underlying diseases are more susceptible to developing severe RSV infections. Chronic lung disease, congenital heart disease, immunodeficiency, and preterm birth (less than 37 weeks gestation) are associated with an increased risk of RSV related ALRI.^48^ Our study found no correlation between RSV disease and underlying diseases and preterm birth, maybe because of the small number of patients with underlying disease. Six out of eight patients who died had underlying diseases, including chronic lung disease, kidney disease, progressive neurological disease, chronic heart disease, and Down's syndrome. Among the demised cases, one was a 7‐month‐old male detected with RSV‐A infection who was admitted to our intensive care unit needing ventilatory support.

Our study found no relationship between RSV severity and prior routine vaccination. We speculate that interrupted routine vaccination and less exposure to respiratory viruses during the COVID‐19 pandemic may have affected the population's immunity against endemic viruses like RSV. This may have delayed regional outbreaks and increased susceptibility to severe disease when pandemic restrictions were lifted.^49^, ^50^ However, there are several controversies due to complex phylogeographic dynamics, viral genotype, and host phenotype interactions in different regions.^49^

Our findings highlight the severity and burden of RSV infection in hospitalized children and underscores the necessity of providing healthcare facilities and efficient preventive and therapeutic approaches. Our study has several limitations. First, the sample size was relatively small and exclusively including hospitalized children. This limits the generalizability of the findings to the broader population as epidemiological information and clinical manifestations of RSV were investigated only in a single center, although the only specialized pediatric center in Golestan province. Second, the prevalence of viral respiratory disease, clinical findings, and the circulation of the virus were not recorded in nonhospitalized children. Third, cases were collected during the winter months, which may have affected the overall prevalence of RSV disease in our region and genotypes detected. Lastly, the molecular technique used to determine the subtypes of the virus was a nested‐PCR, which may not be as accurate as more advanced tests. Therefore, the results of this study should be interpreted with caution and further research is needed to confirm these findings.

## 5 CONCLUSION

In this study, the prevalence of RSV disease in hospitalized children, during the COVID‐19 pandemic, was higher than that of SARS‐CoV‐2 and influenza. The predominant RSV subtype A with circulating genotype ON1 dominated in hospitalized children, whereas subtype B (genotype BA9) was less frequent and not correlated with severity. This aligns with findings from various global studies. Continuous molecular epidemiology studies on respiratory viral infections are vital and beneficial for developing therapeutic strategies and maintaining preparedness against novel outbreaks of viral infections.

## AUTHOR CONTRIBUTIONS

**Mahnaz Ramzali**: Data curation; formal analysis; investigation; writing—original draft. **Vahid Salimi**: Validation; writing—review and editing. **Fatemeh Cheraghali**: Conceptualization; methodology; writing—review and editing. **Seyedeh Delafruz Hosseini**: Formal analysis; investigation; methodology. **Mohammad Yasaghi**: Data curation; formal analysis; investigation. **Saeed Samadizadeh**: Formal analysis; validation; writing—review and editing. **Mostafa Rastegar**: Data curation; software. **Britt Nakstad**: Conceptualization; writing—review and editing. **Alireza Tahamtan**: Conceptualization; methodology; project administration; supervision; validation; visualization; writing—review and editing. All authors have read and approved the final version of the manuscript, had full access to all of the data in this study and take complete responsibility for the integrity of the data and the accuracy of the data analysis.

## CONFLICT OF INTEREST STATEMENT

The authors declare no conflict of interest.

## TRANSPARENCY STATEMENT

The lead author Alireza Tahamtan affirms that this manuscript is an honest, accurate, and transparent account of the study being reported; that no important aspects of the study have been omitted; and that any discrepancies from the study as planned (and, if relevant, registered) have been explained.

## Supporting information


**Supporting information**.

**Supporting information**.

**Supporting information**.

## Data Availability

The authors confirm that the data supporting the findings of this study are available within the article.

## References

[1] Tahamtan A , Samadizadeh S , Rastegar M , Nakstad B , Salimi V . Respiratory syncytial virus infection: why does disease severity vary among individuals? Expert Rev Respir Med. 2020;14(4):415‐423. 31995408 10.1080/17476348.2020.1724095

[2] Tahamtan A , Askari FS , Bont L , Salimi V . Disease severity in respiratory syncytial virus infection: role of host genetic variation. Rev Med Virol. 2019;29(2):e2026. 30609190 10.1002/rmv.2026

[3] Shi T , McAllister DA , O'Brien KL , et al. Global, regional, and national disease burden estimates of acute lower respiratory infections due to respiratory syncytial virus in young children in 2015: a systematic review and modelling study. Lancet. 2017;390(10098):946‐958. 28689664 10.1016/S0140-6736(17)30938-8 PMC5592248

[4] Divarathne M , Ahamed R , Noordeen F . The impact of RSV‐associated respiratory disease on children in Asia. J Pediatr Infect Dis. 2019;14(3):079‐088. 10.1055/s-0038-1637752 PMC7117084 32300274

[5] Tahamtan A , Besteman S , Samadizadeh S , Rastegar M , Bont L , Salimi V . Neutrophils in respiratory syncytial virus infection: from harmful effects to therapeutic opportunities. Br J Pharmacol. 2021;178(3):515‐530. 33169387 10.1111/bph.15318

[6] Salimi V , Tavakoli‐Yaraki M , Yavarian J , Bont L , Mokhtari‐Azad T . Prevalence of human respiratory syncytial virus circulating in Iran. J Infect Public Health. 2016;9(2):125‐135. 26143136 10.1016/j.jiph.2015.05.005

[7] Suryadevara M , Domachowske JB . Epidemiology and seasonality of childhood respiratory syncytial virus infections in the tropics. Viruses. 2021;13(4):696. 33923823 10.3390/v13040696 PMC8074094

[8] Bont L , Checchia PA , Fauroux B , et al. Defining the epidemiology and burden of severe respiratory syncytial virus infection among infants and children in western countries. Infect Dis Ther. 2016;5:271‐298. 27480325 10.1007/s40121-016-0123-0 PMC5019979

[9] Wingert A , Pillay J , Moore DL , et al. Burden of illness in infants and young children hospitalized for respiratory syncytial virus: a rapid review. Can Commun Dis Rep. 2021;47(9):381‐396. 34650335 10.14745/ccdr.v47i09a05 PMC8448381

[10] Tavakoli F , Izadi A , Yavarian J , Sharifi‐Zarchi A , Salimi V , Mokhtari‐Azad T . Determination of genetic characterization and circulation pattern of Respiratory Syncytial Virus (RSV) in children with a respiratory infection, Tehran, Iran, during 2018‐2019. Virus Res. 2021;305:198564. 34530047 10.1016/j.virusres.2021.198564

[11] Alkan Ozdemir S , Soysal B , Calkavur S , et al. Is respiratory syncytial virus infection more dangerous than COVID 19 in the neonatal period? J Matern Fetal Neonatal Med. 2022;35(22):4398‐4403. 33225779 10.1080/14767058.2020.1849125

[12] Mohebi L , Karami H , Mirsalehi N , et al. A delayed resurgence of respiratory syncytial virus (RSV) during the COVID‐19 pandemic: an unpredictable outbreak in a small proportion of children in the Southwest of Iran, April 2022. J Med Virol. 2022;94(12):5802‐5807. 35961780 10.1002/jmv.28065 PMC9538802

[13] Corman VM , Landt O , Kaiser M , et al. Detection of 2019 novel coronavirus (2019‐nCoV) by real‐time RT‐PCR. Euro Surveill. 2020;25(3):2000045. 31992387 10.2807/1560-7917.ES.2020.25.3.2000045 PMC6988269

[14] Kaplan BS , DeBeauchamp J , Stigger‐Rosser E , et al. Influenza virus surveillance in coordinated swine production systems, United States. Emerging Infect Dis. 2015;21(10):1834‐1836. 10.3201/eid2110.140633 PMC4593420 26402228

[15] Audi A , AlIbrahim M , Kaddoura M , Hijazi G , Yassine HM , Zaraket H . Seasonality of respiratory viral infections: will COVID‐19 follow suit? Front Public Health. 2020;8:576. 10.3389/fpubh.2020.567184 PMC7522168 33042956

[16] Malekshahi SS , Razaghipour S , Samieipoor Y , et al. Molecular characterization of the glycoprotein and fusion protein in human respiratory syncytial virus subgroup A: emergence of ON‐1 genotype in Iran. Infect Genet Evol. 2019;71:166‐178. 30946992 10.1016/j.meegid.2019.03.026

[17] Cooney HC , Fleming C , Scheffer IE . Respiratory syncytial virus epidemic during the COVID‐19 pandemic. J Paediatr Child Health. 2022;58(1):215‐216. 34862678 10.1111/jpc.15847

[18] Perez A , Lively JY , Curns A , et al. Respiratory virus surveillance among children with acute respiratory illnesses—New Vaccine Surveillance Network, United States, 2016–2021. MMWR Morb Mortal Wkly Rep. 2022;71(40):1253‐1259. 36201373 10.15585/mmwr.mm7140a1 PMC9541034

[19] Grief SN . Upper respiratory infections. Prim Care. 2013;40(3):757‐770. 23958368 10.1016/j.pop.2013.06.004 PMC7127764

[20] Achangwa C , Park H , Ryu S , Lee M‐S . Collateral impact of public health and social measures on respiratory virus activity during the COVID‐19 pandemic 2020–2021. Viruses. 2022;14(5):1071. 35632810 10.3390/v14051071 PMC9146684

[21] Haddadin Z , Schuster JE , Spieker AJ , et al. Acute respiratory illnesses in children in the SARS‐CoV‐2 pandemic: prospective multicenter study. Pediatrics. 2021;148(2):e2021051462. 33986150 10.1542/peds.2021-051462 PMC8338906

[22] Nunziata F , Salomone S , Catzola A , et al. Clinical presentation and severity of SARS‐CoV‐2 infection compared to respiratory syncytial virus and other viral respiratory infections in children less than two years of age. Viruses. 2023;15(3):717. 36992426 10.3390/v15030717 PMC10055850

[23] Hamid S , Winn A , Parikh R , et al. Seasonality of respiratory syncytial virus—United States, 2017–2023. MMWR Morb Mortal Wkly Rep. 2023;72(14):355‐361. 37022977 10.15585/mmwr.mm7214a1 PMC10078848

[24] Jia R , Lu L , Su L , et al. Resurgence of respiratory syncytial virus infection during COVID‐19 pandemic among children in Shanghai, China. Front Microbiol. 2022;13:938372. 35875547 10.3389/fmicb.2022.938372 PMC9298468

[25] Saravanos GL , Hu N , Homaira N , et al. RSV epidemiology in Australia before and during COVID‐19. Pediatrics. 2022;149(2):e2021053537. 35083489 10.1542/peds.2021-053537

[26] Panatto D , Domnich A , Lai PL , et al. Epidemiology and molecular characteristics of respiratory syncytial virus (RSV) among italian community‐dwelling adults, 2021/22 season. BMC Infect Dis. 2023;23(1):134. 36882698 10.1186/s12879-023-08100-7 PMC9990006

[27] Ujiie M , Tsuzuki S , Nakamoto T , Iwamoto N . Resurgence of respiratory syncytial virus infections during COVID‐19 pandemic, Tokyo, Japan. Emerging Infect Dis. 2021;27(11):2969‐2970. 10.3201/eid2711.211565 PMC8544984 34388086

[28] Chuang Y‐C , Lin K‐P , Wang L‐A , Yeh T‐K , Liu P‐Y . The impact of the COVID‐19 pandemic on respiratory syncytial virus infection: a narrative review. Infect Drug Resist. 2023;16:661‐675. 36743336 10.2147/IDR.S396434 PMC9897071

[29] Halabi KC , Wang H , Leber AL , Sánchez PJ , Ramilo O , Mejias A . Respiratory syncytial virus and SARS‐CoV‐2 coinfections in children. Pediatr Pulmonol. 2022;57:3158‐3160. 35997032 10.1002/ppul.26127 PMC9538042

[30] Kim D , Quinn J , Pinsky B , Shah NH , Brown I . Rates of co‐infection between SARS‐CoV‐2 and other respiratory pathogens. JAMA. 2020;323(20):2085‐2086. 32293646 10.1001/jama.2020.6266 PMC7160748

[31] Kahanowitch R , Gaviria S , Aguilar H , et al. How did respiratory syncytial virus and other pediatric respiratory viruses change during the COVID‐19 pandemic? Pediatr Pulmonol. 2022;57(10):2542‐2545. 35774020 10.1002/ppul.26053 PMC9349531

[32] Swets MC , Russell CD , Harrison EM , et al. SARS‐CoV‐2 co‐infection with influenza viruses, respiratory syncytial virus, or adenoviruses. Lancet. 2022;399(10334):1463‐1464. 35344735 10.1016/S0140-6736(22)00383-X PMC8956294

[33] Kuchar E , Załęski A , Wronowski M , et al. Children were less frequently infected with SARS‐CoV‐2 than adults during 2020 COVID‐19 pandemic in Warsaw, Poland. Eur J Clin Microbiol Infect Dis. 2021;40:541‐547. 32986153 10.1007/s10096-020-04038-9 PMC7520378

[34] Sberna G , Lalle E , Valli MB , Bordi L , Garbuglia AR , Amendola A . Changes in the circulation of common respiratory pathogens among hospitalized patients with influenza‐like illnesses in the Lazio Region (Italy) during fall season of the past three years. Int J Environ Res Public Health. 2022;19(10):5962. 35627498 10.3390/ijerph19105962 PMC9141595

[35] Tempia S , Walaza S , Bhiman JN , et al. Decline of influenza and respiratory syncytial virus detection in facility‐based surveillance during the COVID‐19 pandemic, South Africa, January to October 2020. Euro Surveill. 2021;26(29):2001600. 34296675 10.2807/1560-7917.ES.2021.26.29.2001600 PMC8299743

[36] Alvares PA . SARS‐CoV‐2 and respiratory syncytial virus coinfection in hospitalized pediatric patients. Pediatr Infect Dis J. 2021;40(4):e164‐e166. 33464015 10.1097/INF.0000000000003057

[37] Hashemi SA , Safamanesh S , Ghasemzadeh‐moghaddam H , Ghafouri M , Azimian A . High prevalence of SARS‐CoV‐2 and influenza A virus (H1N1) coinfection in dead patients in Northeastern Iran. J Med Virol. 2021;93(2):1008‐1012. 32720703 10.1002/jmv.26364

[38] Hirsch HH , Martino R , Ward KN , Boeckh M , Einsele H , Ljungman P . Fourth European Conference on Infections in Leukaemia (ECIL‐4): guidelines for diagnosis and treatment of human respiratory syncytial virus, parainfluenza virus, metapneumovirus, rhinovirus, and coronavirus. Clin Infect Dis. 2013;56(2):258‐266. 23024295 10.1093/cid/cis844 PMC3526251

[39] A. Al‐Sharif H , El‐Kafrawy SA , Yousef JM , et al. Dominance of the ON1 genotype of RSV‐A and BA9 genotype of RSV‐B in respiratory cases from Jeddah, Saudi Arabia. Genes. 2020;11(11):1323. 33182267 10.3390/genes11111323 PMC7695323

[40] Chen X , Zhu Y , Wang W , et al. A multi‐center study on molecular epidemiology of human respiratory syncytial virus from children with acute lower respiratory tract infections in the mainland of China between 2015 and 2019. Virologica Sinica. 2021;36(6):1475‐1483. 34398429 10.1007/s12250-021-00430-7 PMC8365132

[41] Tabatabai J , Ihling CM , Rehbein RM , et al. Molecular epidemiology of respiratory syncytial virus in hospitalised children in Heidelberg, Southern Germany, 2014–2017. Infect Genet Evol. 2022;98:105209. 35032683 10.1016/j.meegid.2022.105209

[42] Martinello RA , Chen MD , Weibel C , Kahn JS . Correlation between respiratory syncytial virus genotype and severity of illness. J Infect Dis. 2002;186(6):839‐842. 12198620 10.1086/342414

[43] Tsukagoshi H , Ishioka T , Noda M , Kozawa K , Kimura H . Molecular epidemiology of respiratory viruses in virus‐induced asthma. Front Microbiol. 2013;4:278. 24062735 10.3389/fmicb.2013.00278 PMC3771312

[44] Li W , Wang Y , Yu B , et al. Disease severity of respiratory syncytial virus (RSV) infection correlate to a novel set of five amino acid substitutions in the RSV attachment glycoprotein (G) in China. Virus Res. 2020;281:197937. 32194139 10.1016/j.virusres.2020.197937

[45] Tran DN , Pham TMH , Ha MT , et al. Molecular epidemiology and disease severity of human respiratory syncytial virus in Vietnam. PLoS One. 2013;8(1):e45436. 23349659 10.1371/journal.pone.0045436 PMC3551923

[46] Vianna LA , Siqueira MM , Volpini LPB , Louro ID , Resende PC . Seasonality, molecular epidemiology, and virulence of Respiratory Syncytial Virus (RSV): a perspective into the Brazilian Influenza Surveillance Program. PLoS One. 2021;16(5):e0251361. 34003843 10.1371/journal.pone.0251361 PMC8130917

[47] Ihling CM , Schnitzler P , Heinrich N , et al. Molecular epidemiology of Respiratory Syncytial Virus in children in sub‐Saharan Africa. Trop Med Int Health. 2021;26(7):810‐822. 33683751 10.1111/tmi.13573

[48] Panayiotou C , Richter J , Koliou M , Kalogirou N , Georgiou E , Christodoulou C . Epidemiology of respiratory syncytial virus in children in Cyprus during three consecutive winter seasons (2010–2013): age distribution, seasonality and association between prevalent genotypes and disease severity. Epidemiol Infect. 2014;142(11):2406‐2411. 24476750 10.1017/S0950268814000028 PMC9151279

[49] Garg I , Shekhar R , Sheikh AB , Pal S . Impact of COVID‐19 on the changing patterns of respiratory syncytial virus infections. Infect Dis Rep. 2022;14(4):558‐568. 35893478 10.3390/idr14040059 PMC9394296

[50] Samadizadeh S , Nakstad B , Jamalpoor Z , Tahamtan A . COVID‐19 diagnosis: lessons to learn and hints for preparedness. Expert Rev Mol Diagn. 2022;22(9):851‐853. 36197955 10.1080/14737159.2022.2132852

