# Supplementary figures and images for: Epidemiology and clinical features of respiratory syncytial virus (RSV) infection in hospitalized children during the COVID‐19 pandemic in Gorgan, Iran
Source: Health Sci Rep. 2024 Jan 3;7(1):e1787. doi: 10.1002/hsr2.1787 (PMC10764657; doi:10.1002/hsr2.1787)

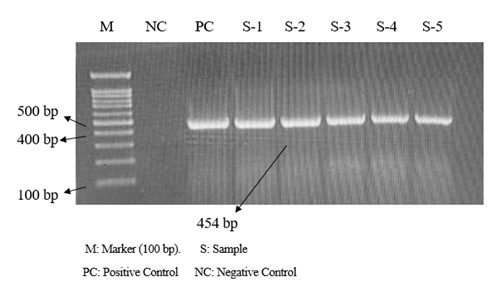

Supplement: Supplementary file 1 — Supporting information. [file HSR2-7-e1787-s001.tif]

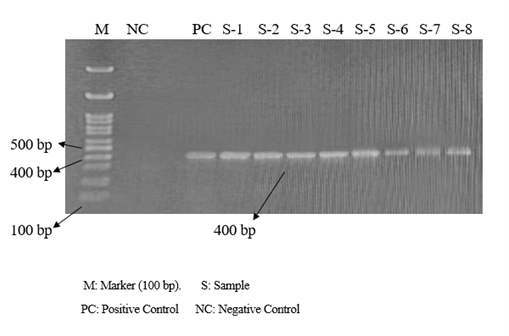

Supplement: Supplementary file 2 — Supporting information. [file HSR2-7-e1787-s002.tif]

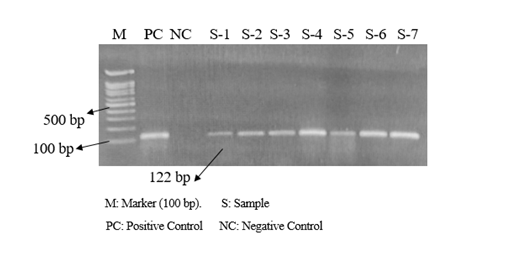

Supplement: Supplementary file 3 — Supporting information. [file HSR2-7-e1787-s003.tif]
